# High-Performance Layered CaV_4_O_9_-MXene Composite Cathodes for Aqueous Zinc Ion Batteries

**DOI:** 10.3390/nano13091536

**Published:** 2023-05-03

**Authors:** Luan Fang, Li Lin, Zhuomei Wu, Tianhao Xu, Xuxu Wang, Limin Chang, Ping Nie

**Affiliations:** Key Laboratory of Preparation and Applications of Environmental Friendly Material of the Ministry of Education & College of Chemistry, Jilin Normal University, Changchun 130103, China

**Keywords:** aqueous, zinc ion batteries, vanadium-based cathode, CaV_4_O_9_, MXene

## Abstract

Due to their reliability, affordability and high safety, rechargeable aqueous zinc ion batteries (ZIBs) have garnered a lot of attention. Nevertheless, undesirable long-term cycle performance and the inadequate energy density of cathode materials impede the development of ZIBs. Herein, we report a layered CaV_4_O_9_-MXene (Ti_3_C_2_T*_x_*) composite assembled using CaV_4_O_9_ nanosheets on Ti_3_C_2_T_x_ and investigate its electrochemical performance as a new cathode for ZIBs, where CaV_4_O_9_ nanosheets attached on the surface of MXene and interlamination create a layered 2D structure, efficiently improving the electrical conductivity of CaV_4_O_9_ and avoiding the stacking of MXene nanosheets. The structure also enables fast ion and electron transport. Further discussion is conducted on the effects of adding MXene in various amounts on the morphology and electrochemical properties. The composite shows an improved reversible capacity of 274.3 mA h g^−1^ at 0.1 A g^−1^, superior rate capabilities at 7 A g^−1^, and a high specific capacity of 107.6 mA h g^−1^ can be delivered after 2000 cycles at a current density of 1 A g^−1^. The improvement of the electrochemical performance is due to its unique layered structure, high electrical conductivity, and pseudo capacitance behavior.

## 1. Introduction

The energy crisis and environmental pollution make the development of large-scale energy storage systems imminent. Lithium-ion batteries (LIBs) dominate the energy storage field of 3C electronics and the electric vehicle industry [1,2,3,4]. Nevertheless, the limited resources of lithium, safety, and high cost dramatically hinder the future sustainability of lithium-ion batteries [5,6,7]. Recently, aqueous zinc ion batteries (ZIBs) with lower cost, higher security, high efficiency, and ultra-high theory capacity (820 mAh g^−1^) have attracted increasing attention [8,9,10]. Yet, achieving high performance aqueous ZIBs with long-term life cycles and high energy density remains a challenge owing to the low electrical conductivity and structural instability of conventional cathode materials [11,12,13,14,15]. Currently, the widely reported cathodes for ZIBs are mainly based on manganese- or vanadium-based oxides, Prussian blue analogs, spinel-structured oxides, and organic materials [5,16,17,18,19]. Among those materials, vanadium-based materials show promising because of their tremendous natural richness, multiple valence states, and unique layered structure.

Although vanadium-based materials have made great progress in the intercalation/deintercalation of Zn^2+^, such materials often suffer from slow diffusion kinetics, irreversible phase transitions, and even structural collapse [20,21]. One of the most effective strategies is the introduction of metal ions such as M_x_V_2_O_5_·nH_2_O (M = Zn, Ca, Mg). Metal ions in vanadium oxides can enhance the structural stability of the host material, and thus improve the cyclic performance [22,23]. Calcium vanadate (CaV_4_O_9_, CVO), having a typical lamellar structure in which ${\left[V_{4}O_{9}\right]}_{n}^{2n-}$ sheets consist of a VO_5_ square pyramid with shared vertices, shows higher conductivity and specific surface area [20,24,25,26]. The strength of the V–O bond is large, can effectively mitigate structural stress change induced by the ion insertion process, and enhance the electrochemical performance of the material. Therefore, CaV_4_O_9_ is widely used in lithium-ion/sodium-ion batteries. Wu et al. applied CaV_4_O_9_ as an anode material in lithium-ion batteries [27]; the electrode showed both a high capacity of more than 600 mAh g^−1^ with a safe average discharge potential of about 0.8 V and near-zero volume change characteristics. Xu et al. reported CaV_4_O_9_ nanowires in sodium-ion batteries [28], and the prepared electrodes delivered high reversible capacity, excellent cycling, and multiplicity performance. 

However, the utilization of CaV_4_O_9_ in zinc ion batteries has rarely been reported. Recently, Du et al. prepared CaV_4_O_9_/CNTs composite film as the cathode for ZIBs using a consecutive spray printing technique [29]. Ex situ XRD and XPS analysis results demonstrated that the generation of amorphous V_2_O_5_·nH_2_O during charging, which can provide more ion channels, speeds up charge transfer at the electrode/electrolyte interface. Huang et al. reported an electrochemically induced in situ transformation method to synthesize oxygen-deficient navajoite (V_5_O_12−*x*_·6H_2_O, HVO_d_) covered by gypsum (CaSO_4_·2H_2_O, GP) layers using CaV_4_O_9_ as the pristine cathode material. GP facilitates the desolvation of hydrogenated zinc ions through its hydrophilic surface and constrained tunneling [30]. Wu et al. prepared novel hydrated vanadate (CaV_8_O_20_·3H_2_O) nanoribbons with graphene as the cathode material for aqueous zinc ion batteries [31]. Ca^2+^ and crystal water as columns enhance the stability of the framework and reduce electrostatic interactions with Zn^2+^. However, calcium vanadate materials tend to aggregate, showing unsatisfactory cycling stability, low specific capacity (<430 mA h g^−1^), and their charge storage capacity is much lower than the theoretical capacity of V_2_O_5_ (585 mA h g^−1^). To address these issues, compositing conductive material alleviates the volume expansion. As an attractive 2D layered metal carbide/nitride, MXenes have been considered as ideal conductive materials for developing composite electrodes with high-rates due to their unique 2D structure, abundant surface functional groups, high electrical conductivity, and good hydrophilicity [32,33,34].

Herein, we report the reasonable design of the hierarchical assembled CaV_4_O_9_-MXene (Ti_3_C_2_T_x_) layered composite via a solvothermal method. CaV_4_O_9_ nanosheets are homogeneously loaded onto the interlayer and surface of MXene, forming a unique layered structure. The morphology can improve the conductivity of the nanocomposites. Furthermore, the strong interaction between grown CaV_4_O_9_ nanosheets and MXene substrates promotes fast ion insertion/extraction of kinetics and structure stability. Investigations are also conducted into how different amounts of MXene affected the zinc ion storage performance. The pseudocapacitive behavior of CaV_4_O_9_-Ti_3_C_2_T_x_ is further analyzed, playing an essential role in the specific capacity contribution. Due to the benefits of the unique structural characteristics, CaV_4_O_9_-MXene-0.1 exhibits an enhanced specific capacity of 274.3 mAh g^−1^ at 0.1 A g^−1^, superior rate capabilities, and long cycling stability, a high specific capacity of 107.6 mAh g^−1^ can be delivered after 2000 cycles at a current density of 1 A g^−1^.

## 2. Experimental Methods

### 2.1. Materials Synthesis

#### 2.1.1. Synthesis of Ti_3_C_2_T_x_ MXene

Firstly, 2 g of Ti_3_AlC_2_ MAX powder was placed in a plastic beaker containing 20 mL of HF (49 wt%), stirred at ambient temperature for 24 h to etch the Al element in the MAX, then the sample was washed with distilled water to pH = 7 and dried in vacuum at 60 °C for 12 h to collect Ti_3_C_2_T*_x_* MXene powder.

#### 2.1.2. Synthesis of CaV_4_O_9_-MXene

1 mmol of Ca(OH)_2_ was added to a mixture of 10 mL glycerol and 10 mL water, 2 mmol V_2_O_5_ was dispersed in 10 mL of deionized water, then 5 mL H_2_O_2_ solution was slowly added. Stirring the two solutions separately for 1 h, afterwards it was stirred for 2 h after mixing; finally, MXene powder was added and stirred for another 2 h. The mixture was placed in a 50 mL polytetrafluoroethylene kettle encapsulated in a stainless-steel reactor and kept at 200 °C for 48 h. After solvothermal reaction, the CaV_4_O_9_-MXene material was obtained using suction filtration, repeated cleaning with distilled water and ethanol, and the material was dried in vacuum for 12 h. For comparison, the samples with different amounts of MXene, including 0.1 g, 0.2 g, 0.3 g, were prepared, and the obtained samples were labeled as CaV_4_O_9_-MXene-0.1, CaV_4_O_9_-MXene-0.2, and CaV_4_O_9_-MXene-0.3, respectively.

### 2.2. Characterizations

The prepared products were morphologically analyzed using field emission scanning electronic microscopy (FESEM, JSM-7800F) and transmission electronic microscopy (TEM, JEM-2100, JEOL, Tokyo, Japan). XRD pattern was performed with a powder X-ray diffraction system (XRD, Rigaku d/max PC2500, Tokyo, Japan) with Cu Kα in a range from 5° to 90°. Nitrogen adsorption/desorption isotherms were determined with an Isorb-HP2 analyzer (Quantachrome Instruments, Boynton Beach, FL, USA) at 77 K with liquid nitrogen. X-ray photoelectron spectrometry (XPS) was measured on a Thermo Scientific (Waltham, MA, USA) ESCALAB 250Xi spectrometer.

### 2.3. Electrochemical Measurements

To prepare the CaV_4_O_9_-MXene cathode, the electrochemical active material (CaV_4_O_9_-MXene) was mixed with ethynyl black and polyphenylene fluoride (PVDF) in N-methyl pyrrolidone in a ratio of 8:1:1 by weight, then the slurry was coated on titanium foil polished with sandpaper, while zinc foil and a glass fiber membrane (GF/D) were applied as the anode and separator, respectively. Deoxygenated 3M Zn (CF_3_SO_3_)_2_ was used as the electrolyte. Subsequently, the cathode was dried in vacuum under 60 °C for 12 h. CR2032 cells were fabricated using as-prepared electrodes in air. The electrochemical properties of the cells were evaluated using a LAND battery test system (CT2001A), including specific capacity, rate performance, and long-term cycling stability. Cyclic voltammetry (CV) measurements were performed on a CHI 760E electrochemical workstation. Contact angle test was performed on a JC 2000D1 contact Angle tester.

## 3. Results and Discussion

### 3.1. Structure Characterization

**Figure 1:**
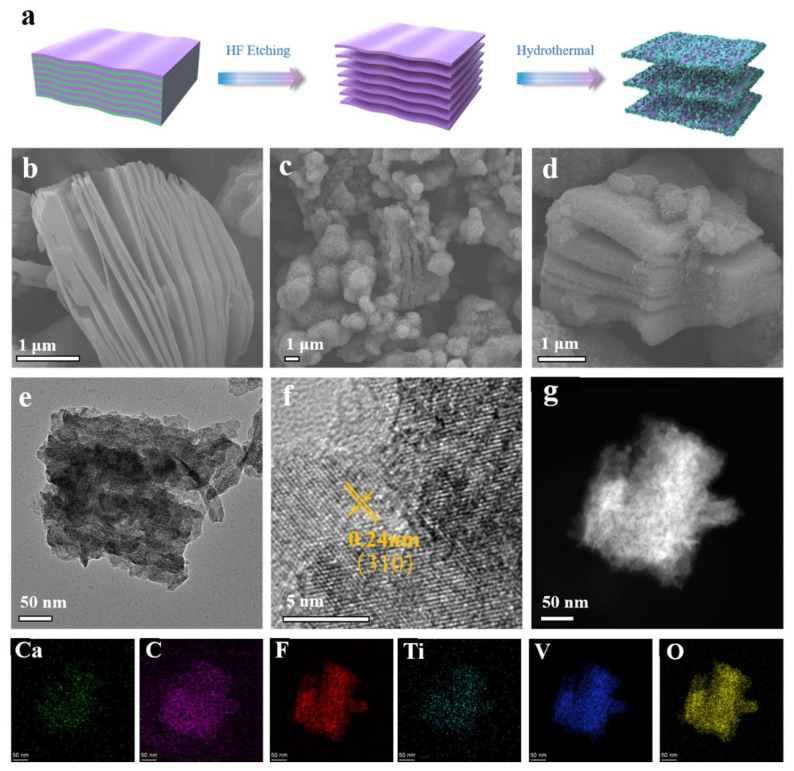
(**a**) Schematic illustration showing the synthesis process of CaV_4_O_9_-MXene composite, (**b**) SEM images of MXene, (**c**,**d**) SEM images of CaV_4_O_9_-Mxene-0.1, (**e**,**f**) HRTEM images of CaV_4_O_9_-Mxene-0.1, and (**g**) STEM image and EDS elemental mappings of Ca, C, F, Ti, V, and O of the CaV_4_O_9_-Mxene-0.1.

Figure 1a depicts the preparation process of CaV_4_O_9_-MXene composite. Firstly, MXene was synthesized via a hydrogen fluoride solution etching Al atom from a MAX phase. CaV_4_O_9_ nanosheets homogeneously anchored on MXene surface were obtained using a simple solvothermal strategy. We prepared three samples (CaV_4_O_9_-MXene-0.1, CaV_4_O_9_-MXene-0.2, and CaV_4_O_9_-MXene-0.3) with different MXene contents based on the mass of MXene used (0.1 g, 0.2 g, and 0.3 g). Figure 1b–d and Figure S1 displays scan electron microscope (SEM) images of MXene and CaV_4_O_9_-MXene materials. As can be seen from Figure 1c, CaV_4_O_9_ nanosheets grew uniformly on the MXene surface and were able to preserve the layer structure of MXene; the morphology can enlarge the layer intervals of MXene and increase the surface area and sites for ion storage. In addition, CaV_4_O_9_ consists of numerous interleaved ultra-thin nanosheets forming a spherical flower structure, which may be due to the absence of MXene substrate. In contrast, composites with a higher concentration of MXene exhibited a stacked layered structure and full coating (Figure S1a–d).

The morphology of CaV_4_O_9_-MXene-0.1 was investigated using high-resolution transmission electron microscopy (HRTEM). As shown in Figure 1e–g, it appears that the presence of ultrathin CaV_4_O_9_ nanosheets uniformly anchored on Ti_3_C_2_T*_x_* layers. The crystalline lattice of CaV_4_O_9_-MXene-0.1 can be clearly seen in Figure 1f**.** The crystal lattice distance was 0.24 nm, indexed to the (310) crystal plane of CaV_4_O_9_ [26]. Energy dispersive X-ray spectroscopy (EDS) element mapping further revealed the uniform distribution of calcium, carbon, fluorine, titanium, vanadium, and oxygen elements in CaV_4_O_9_-MXene-0.1 material (Figure 1g).

**Figure 2:**
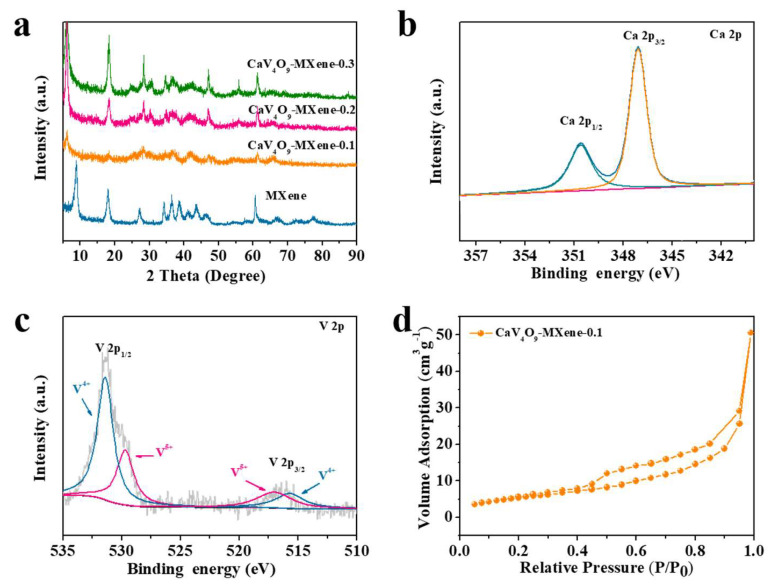
(**a**) XRD patterns of CaV_4_O_9_-MXene samples, (**b**) XPS spectra of CaV_4_O_9_-Mxene-0.1 composite: Ca 2p region, (**c**) V 2p region, (**d**) nitrogen adsorption/desorption isotherms of CaV_4_O_9_-Mxene-0.1.

To explore the microstructure of the CaV_4_O_9_-MXene cathode material synthesized under different amounts of MXene, XRD patterns were recorded (Figure 2a). From the XRD patterns, we found that the characteristic peaks of composites appeared at 29.8°, 33.7°, 43.5°, and 48.8°, respectively, and no impurity was detected, showing that CaV_4_O_9_ is a purified phase [35]. The composite showed weak MXene diffraction peaks compared with pure MXene, which may be due to the low MXene content and the coating of CaV_4_O_9_ nanosheets on the MXene surface. X-ray photoelectron spectroscopy (XPS) was used to analyze the surface chemical properties and chemical states of CaV_4_O_9_-MXene-0.1. Based on the full spectrum of CaV_4_O_9_-MXene-0.1, peaks at 346.8 eV, 517.4 eV, and 531.0 eV correspond to Ca 2p, V 2p, and O 1s, respectively (Figure S2). As shown in Figure 2b, the Ca 2p spectrum showed two types of bands at 347.5 eV and 350.8 eV [36]. In the high-resolution V 2p spectrum (Figure 2c), CaV_4_O_9_-MXene-0.1 showed the characteristic peak attributed to V^4+^ at 515.7 and 531.4 eV, and the dominant V^5+^ signal at 517.2 and 529.7 eV [37]. The surface area of CaV_4_O_9_-Mxene-0.1 was measured using the N_2_ adsorption/desorption curves (Figure 2d). The results showed that the specific surface area of the composite was 20.315 m^2^ g^−1^ due to the uniform coating of CaV_4_O_9_ nanoflakes on the surface of MXene. The adsorption and desorption curves showed that the CaV_4_O_9_-MXene-0.1 composite had H_3_ hysteresis loops in the type IV isotherm [6,38], indicating mesoporous properties of the composites.

### 3.2. Electrochemical Performance

**Figure 3:**
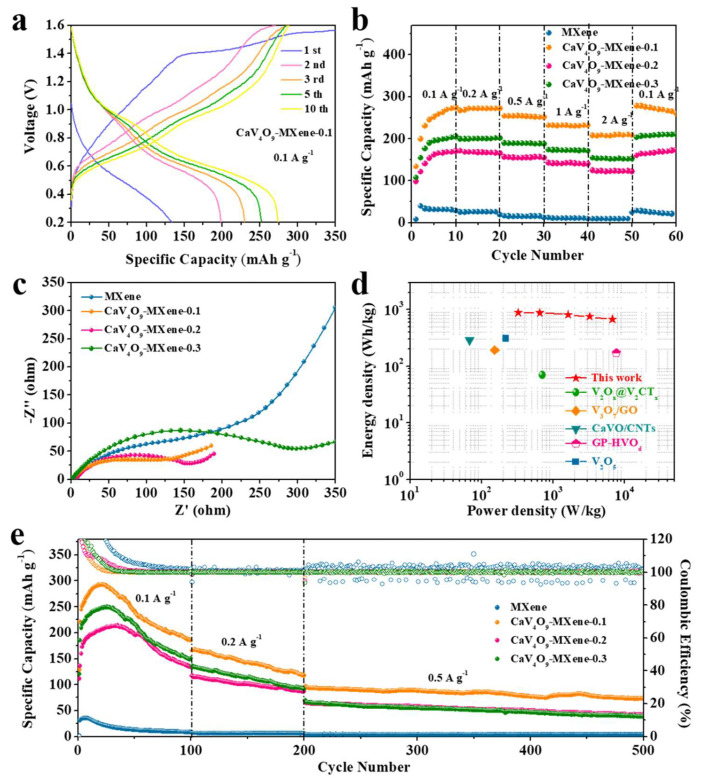
Electrochemical properties of CaV_4_O_9_-MXene composites as cathodes for AZIB: (**a**) galvanostatic charge/discharge curves of CaV_4_O_9_-Mxene-0.1 at 0.1 A g^−1^ in 0.2–1.6 V, (**b**) rate capability of CaV_4_O_9_-MXene composite from 0.1 to 2 A g^−1^, (**c**) EIS of MXene and CaV_4_O_9_-MXene composites, (**d**) the Ragone plots of CaV_4_O_9_-MXene compared with other reported cathode materials in aqueous ZIBs, (**e**) cyclic performance of CaV_4_O_9_-MXene composite at different current densities.

To identify the distinctive structural benefits of CaV_4_O_9_-MXene material, electrochemical tests were carried out. Figure S3 shows the galvanostatic charge–discharge (GCD) curve of MXene at a current density of 0.1 A g^−1^. As can be seen, the stable discharge capacity was only 39 mAh g^−1^, and after compositing with CaV_4_O_9_, a higher specific capacity was obtained (Figure 3a and Figure S4). By comparing the composites with different proportions, CaV_4_O_9_-MXene-0.1 achieved a higher specific discharge capacity of 274.3 mAh g^−1^ after 10 cycles, which means that the composite may be helpful to improve the zinc storage capacity of CaV_4_O_9_-MXene material. Notably, the low Coulombic efficiencies of the first cycle correspond to electrolyte decomposition and SEI film formation [39]. The rate performance of the MXene and CaV_4_O_9_-MXene cathode is compared in Figure 3b at different current densities from 0.1 to 2 A g^−1^. Taking CaV_4_O_9_-MXene-0.1 as an example, the discharge capacities of 271.6 mAh g^−1^, 267.2 mAh g^−1^, 254.2 mAh g^−1^, 232.1 mAh g^−1^, and 207.5 mAh g^−1^ can be achieved at current densities of 0.1, 0.2, 0.5, 1, and 2 A g^−1^, respectively. While the current recovered back to 0.1 A g^−1^, the reversible capacity was covered to 260.1 mAh g^−1^, corresponding to a capacity retention of 95.8%. Similarly, for CaV_4_O_9_-MXene-0.2 and CaV_4_O_9_-MXene-0.3, as the current rate recovered after 50 cycles to 0.1 A g^−1^, the discharge capacity was 159.6 and 202.6 mAh g^−1^, respectively. Obviously, compared with pure MXene, the composite material exhibited improved electrochemical properties, among which the performance of CaV_4_O_9_-MXene-0.1 was the best, in accordance with the results of galvanostatic charge/discharge test.

In comparison with pure MXene, all composites displayed improved cycling properties as shown in Figure 3e. When the current density was 0.1 A g^−1^, the specific discharge capacity of CaV_4_O_9_-MXene-0.1 remained at 185.1 mA h g^−1^ after 100 cycles. We continued to carry out the cycle performance test at 0.2 A g^−1^. After 100 cycles, the specific discharge capacity was still 117.4 mA h g^−1^. When we used a higher current density of 0.5 A g^−1^, the specific discharge capacity was 73.2 mA h g^−1^ after 300 cycles. This shows that the CaV_4_O_9_-MXene composite exhibits good stability. The electrolyte ion and electron transport characteristics of MXene and the CaV_4_O_9_-MXene composite were further analyzed using electrochemical impedance spectroscopy (EIS) (Figure 3c). The semicircle observed in the high frequency area responds to the charge transfer (Rct) impedance at the interface [40]. The *R*ct value of CaV_4_O_9_-MXene was significantly lower than that of MXene, indicating faster charge transfer in electrochemical reactions. For comparison, CaV_4_O_9_-MXene-0.1 showed smaller impedance and better conductivity, indicating the faster kinetics characteristics of CaV_4_O_9_-MXene over MXene. The Ragone plots are shown in Figure 3d; the delivered specific energy and power density of CaV_4_O_9_-MXene was 888.79 Wh kg^−1^ at 325.56 W kg^−1^, and 675.93 Wh kg^−1^ at 6759.3 W kg^−1^, which is superior than other reported cathodes in aqueous ZIBs, such as V_2_O*_x_*@V_2_CT*_x_* (70 Wh kg^−1^, 705.6 W kg^−1^) [34], V_3_O_7_/GO (191.8 Wh kg^−1^, 153.4 W kg^−1^) [41], CaVO/CNTs (290 Wh kg^−1^, 68 W kg^−1^) [29], GP-HVO_d_ (173 Wh kg^−1^, 7688 W kg^−1^) [42], and V_2_O_5_ (310.3 Wh kg^−1^, 217.8 W kg^−1^) [43].

**Figure 4:**
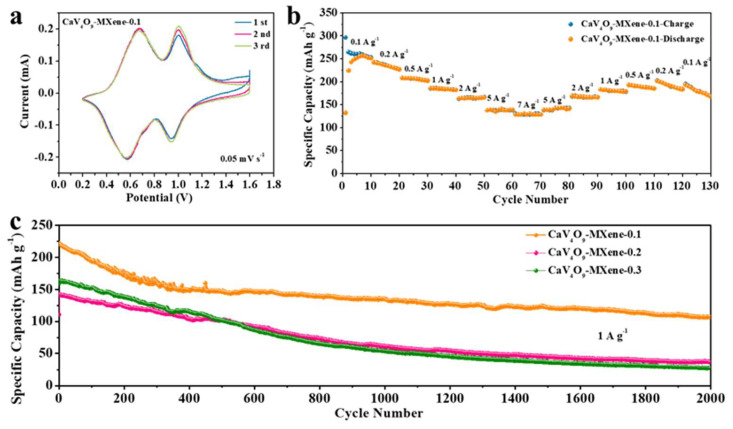
(**a**) Typical CV curves of CaV_4_O_9_-Mxene-0.1 recorded with a scan rate of 0.05 mV s^−1^, (**b**) rate capability of CaV_4_O_9_-Mxene-0.1 composite from 0.1 to 7 A g^−1^, (**c**) cyclic performance of CaV_4_O_9_-MXene composite at 1 A g^−1^.

To determine the reaction mechanism, cyclic voltammetry (CV) curves were obtained in initial several cycles at a scan rate of 0.05 mV s^−1^ in the voltage range of 0.2–1.6 V. As shown in Figure 4a, the CV curve showed two pairs of distinct redox peaks. The oxidation peaks were located at 0.671 V and 1.001 V, and the reduction peaks were at 0.577 V and 0.94 V, respectively. It is proved that there is a two-step reaction process in the insertion/extraction process of Zn^2+^ [44]. In the following scan, the CV curve had a good degree of coincidence, and the redox peak of CaV_4_O_9_-MXene-0.1 was almost unchanged, which proves good stability and reversibility. At high current density (Figure 4b), the CaV_4_O_9_-MXene-0.1 still exhibited an excellent capacity retention. When the current density ranged from 0.1 to 7 to 0.1 A g^−1^, the CaV_4_O_9_-MXene-0.1 electrode showed excellent rate performance, and CaV_4_O_9_-MXene-0.1 provided higher reversible capacities of 246.6, 185.5, 164.1, 137.4, and 130.2 mA h g^−1^ at 0.1, 1, 2, 5, and 7 A g^−1^, respectively. When the current gradually recovered to 0.1 A g^−1^, 77.1% of the initial capacity was restored, indicating that MXene can synergistically accelerate kinetics and enhance the rate capability. Figure 4c is the comparison of cycle performance at a current density of 1 A g^−1^. The specific discharge capacity of CaV_4_O_9_-MXene-0.1 in the first cycle was 221.6 mA h g^−1^, which can be stably cycled for 2000 times. The cycling performances of the CaV_4_O_9_-MXene-0.2 and CaV_4_O_9_-MXene-0.3 composites showed obvious attenuation, and CaV_4_O_9_-MXene-0.1 showed the best cycling stability. The rate and cycling performance of CaV_4_O_9-_MXene were superior to many reported vanadium-based cathodes (Table S1).

### 3.3. Electrochemical Kinetics

**Figure 5:**
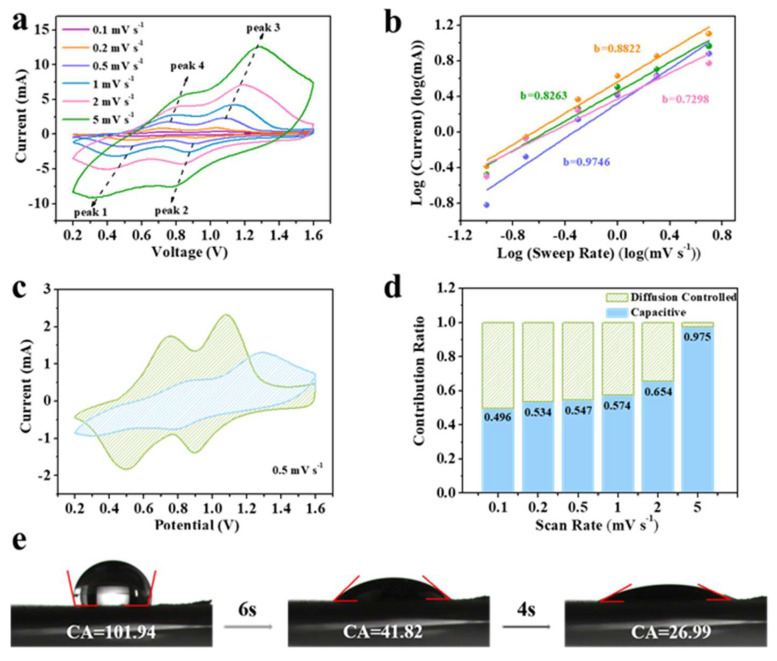
(**a**) CV profiles at various scan rates for CaV_4_O_9_-Mxene-0.1, (**b**) *b* values of anode and cathode peak currents determined by fitting log (*i*) vs. log (ν), (**c**) CV at 0.5 mV s^−1^, blue shaded area indicating capacitance contribution, (**d**) ratio of the contribution of capacitance and diffusion behavior at different scan rates, (**e**) contact angles of an electrolyte droplet on CaV_4_O_9_-Mxene-0.1 sample.

To further reveal the electrochemical kinetics of the zinc ion diffusion process and the contribution of pseudo capacitance behavior of the CaV_4_O_9_-MXene-0.1 electrode, CV curves in the voltage range of 0.2–1.6 V at different scan rates are provided in Figure 5a. All CV curves showed two pairs of redox peaks with similar shape, which correspond to the voltage plateau of the charge–discharge curves and can be attributed to a two-step reaction of Zn^2+^ in the CaV_4_O_9_-MXene lattice. Generally, the relationship between peak current (*i*) and scan rate (v) can be expressed as:

(1) $$i=av^{b}$$

(2) $$\log\left(i\right)=blog\left(v\right)+log(a)$$

where i and v are current and scan rate, and a and b are variable parameters. In Formula (2), the slopes of log (i) and log (v) can be used to calculate the value of b, which can analyze the charge storage mechanism during electrochemical reactions [45]. When the electrode process is controlled by diffusion, the value of b is 0.5, manifesting as the response current proportionating to the square root of the voltage scan rate. When the electrode process is controlled by capacitance, the value of b is one. As shown in b, the *b* values of peaks P1, P2, P3, and P4 are 0.83, 0.97, 0.88, and 0.73, respectively, demonstrating that the electrode is mainly controlled by the pseudo capacitance and, thus, has a fast zinc storage performance. In addition, the potential-dependent capacitive behavior is identified by the previously reported method:

(3) $$i=k_{1}v+k_{2}v^{1/2}$$

where k is a constant and the responding current (i) at a given voltage (V) is quantified as $k_{1}$ν (capacitive effect) and $k_{2}$ν^1/2^ (diffusion control behavior) [11]. The equation can be transferred into the format below:

(4) $$i/v^{1/2}=k_{1}v^{1/2}+k_{2}$$

Therefore, it can be analyzed based on the linear relationship between $i/v^{1/2}$ and $v^{1/2}$. From Figure 5c, the contribution of pseudo capacitance was about 54.7% at a scan speed of 0.5 mV s^−1^. As shown in Figure 5d, the contribution rate of pseudo capacitance at different scan rates was obtained. When the scan rate was 0.1, 0.2, 0.5, 1, 2, and 5 mV s^−1^, the contribution was 49.6, 53.4, 54.7, 57.4, 65.4, and 97.5%, respectively. The high pseudo capacitance control is helpful to accelerate the charging and discharging rate. A high percentage of capacitive behavior can bring fast response kinetics of the electrodes, which contributes to its high-rate properties. Furthermore, the contact angle of CaV_4_O_9_-MXene-0.1 after dropping with 2 μL electrolyte reached 101.94° immediately, and subsequently reduced to 26.99° after 10 s, indicating good wettability (Figure 5e). This facilitated the reduction in resistance and fast Zn^2+^ transfer.

## 4. Conclusions

In summary, we designed and synthesized a CaV_4_O_9_-MXene composite through an efficient solvothermal strategy. CaV_4_O_9_ nanosheets were uniformly anchored on the layer and surface of MXene, which expanded the MXene layer. The expanded specific surface area provided abundant active sites for Zn^2+^ storage, and the addition of MXene enhanced the electrical conductivity. Benefiting from the synergistic effect of enhanced electron/ion transfer and a unique layered structure, the as-obtained CaV_4_O_9_-MXene exhibited an excellent cycling and rate performance when used as a cathode for AZIBs. Specifically, the CaV_4_O_9_-MXene-0.1 cathode exhibited a highly reversible capacity of 107.6 mAh g^−1^ after 2000 cycles at a current density of 1 A g^−1^. It showed good rate performance with a specific capacity of 142.9 mAh g^−1^, even at a high current density of 7 A g^−1^. Additionally, it had excellent structural stability, high energy density, and power density (888.79 Wh kg^−1^ at 325.56 W kg^−1^). This work provides a method for the design of high-performance electrode materials featuring layered MXene and vanadium-based materials, and creates a novel pathway for the application of low-cost ZIBs systems.

## Data Availability

The data presented in this study are available on request from the corresponding author.

## Supplementary Materials

The following supporting information can be downloaded at: https://www.mdpi.com/article/10.3390/nano13091536/s1, Figure S1: (a,b) SEM images of CaV_4_O_9_-MXene-0.2 and CaV_4_O_9_-MXene-0.3 (c,d); Figure S2: XPS spectrum of the CaV_4_O_9_-MXene-0.1 composite; Figure S3: Galvanostatic charge/discharge curves of MXene at 0.1 A g^−1^; Figure S4: (a,b) Galvanostatic charge/discharge curves of CaV_4_O_9_-MXene-0.2 and CaV_4_O_9_-MXene-0.3 at 0.1 A g^−1^, respectively; Table S1: The comparison of the rate and cycling performance of vanadium-based cathodes in aqueous ZIBs. References [46,47,48,49,50,51,52,53] are cited in the supplementary materials.
