# Supplementary material for: High-Performance Layered CaV4O9-MXene Composite Cathodes for Aqueous Zinc Ion Batteries
Source: Nanomaterials (Basel). 2023 May 3;13(9):1536. doi: 10.3390/nano13091536 (PMC10180448; doi:10.3390/nano13091536)
Supplement: Supplementary file 1 [file nanomaterials-13-01536-s001.zip › nanomaterials-2331505-supplementary.pdf]

nanomaterials

MDPI

Supplementary Materials

High-Performance Layered CaV_4O_9-MXene Composite Cathodes for Aqueous Zinc Ion Batteries

Luan Fang †, Li Lin †, Zhuomei Wu, Tianhao Xu *, Xuxu Wang, Limin Chang * and Ping Nie

Key Laboratory of Preparation and Applications of Environmental Friendly Material of the Ministry of Education & College of Chemistry, Jilin Normal University, Changchun 130103, China; fangluan9194@163.com (L.F.); 18843425325@163.com (L.L.); wuzhuomei1998@163.com (Z.W.); wangxx@jlnu.edu.cn (X.W.); xdnieping2009@sina.com (P.N.)

* Correspondence: aaaa2139@163.com (L.C.); 1808016@jlnu.edu.cn (T.X.)

† These authors contributed equally to this work.

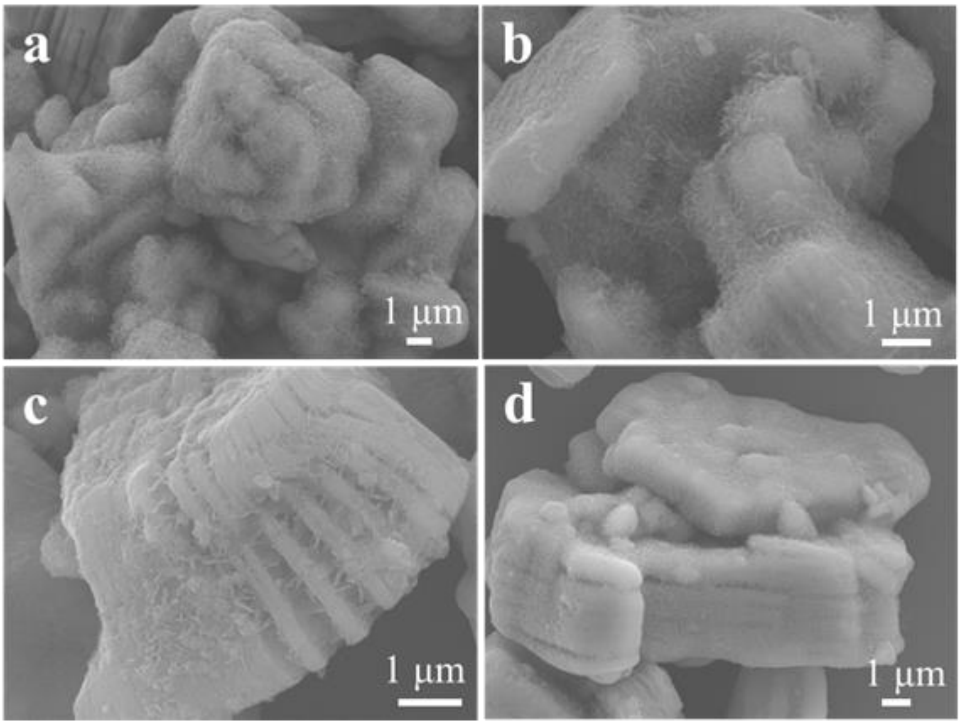

Figure S1. (**a**,**b**) SEM images of CaV_4O_9-MXene-0.2, CaV_4O_9-MXene-0.3 (**c**,**d**).

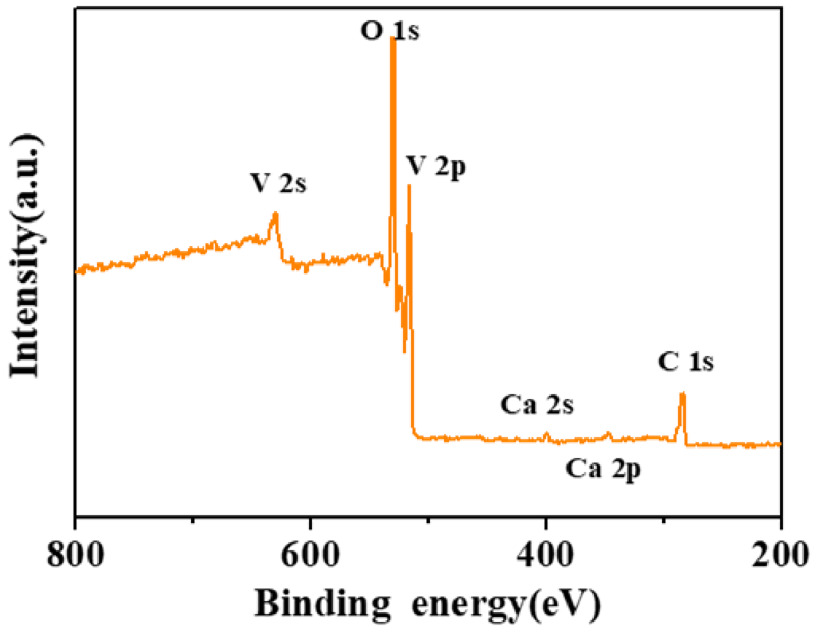

Figure S2. XPS spectrum of the CaV_4O_9-MXene-0.1 composite.

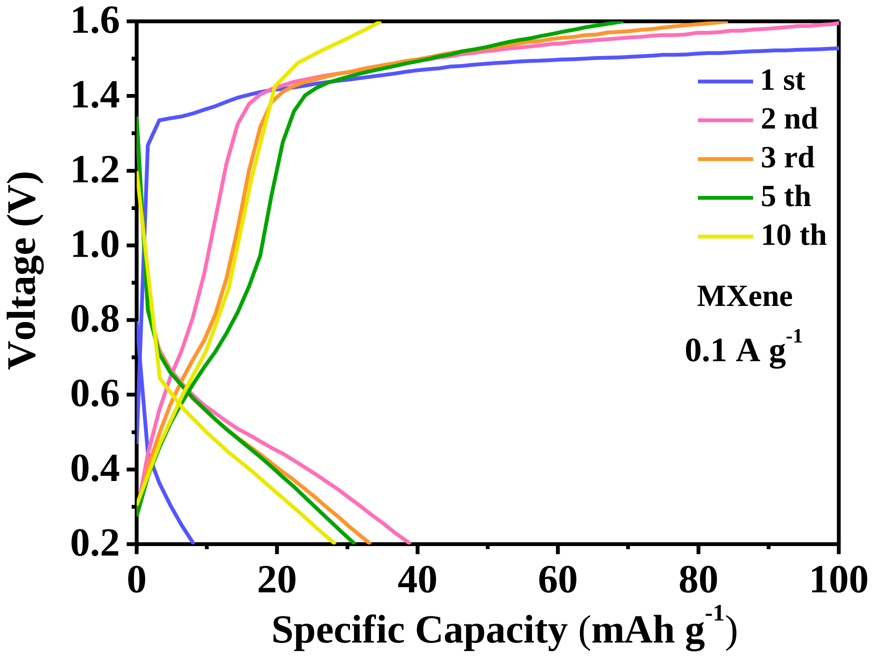

Figure S3. Galvanostatic charge/discharge curves of MXene at 0.1 A g^{-1}.

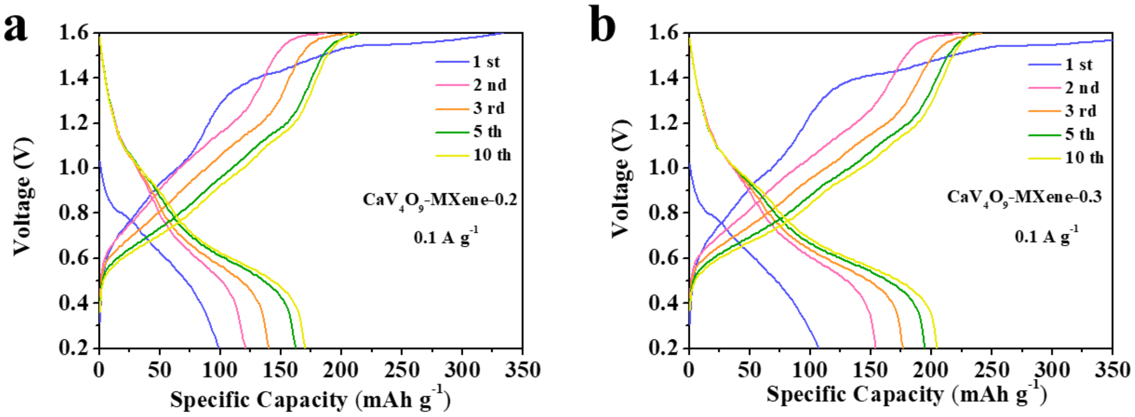

Figure S4. (**a**, **b**) Galvanostatic charge/discharge curves of CaV_4O_9-MXene-0.2 and CaV_4O_9-MXene-0.3 at 0.1 A g^{-1}, respectively.

Table S1. The comparison of the rate and cycling performance of vanadium-based cathodes in aqueous ZIBs.

Cathode	Electrolyte	Rate performance	Cycling performance	Ref.
**CaV_4O_9-MXene**	3M $Zn(CF_3SO_3)_2$	246.6, 185.5, 164.1, 137.4 and 130.2 mAh g^{-1} at 0.1, 1, 2, 5 and 7 A g^{-1}, respectively	107.6 mAh g^{-1} after 2000 cycles at 1000 mA g^{-1}	This work
**V_2O_5**	3 M $ZnSO_4$	80% retention at 100 mA g^{-1}	78.92% after 1000 cycles at 1000 mA g^{-1}	[46]
**Layered VS_2**	1 M $ZnSO_4$	159.1 mAh g^{-1} at 100 mA g^{-1}, 136.8 mAh g^{-1} at 500 mA g^{-1}	110.9 mAh g^{-1} after 200 cycles at 500 mA g^{-1}	[47]
**$V_2O_5{\cdot}nH_2O$/MXene**	3M $Zn(CF_3SO_3)_2$	262 mAh g^{-1} at 1 A g^{-1}, 225 mAh g^{-1} at 2 A g^{-1}	223 mAh g^{-1} after 50 cycles at 100 mA g^{-1}	[48]
**$Ca_{0.25}V_2O_5{\cdot}nH_2O$**	1 M $ZnSO_4$	85% retention at 0.2C	96% after 3000 cycles at 80C	[49]
**$NH_4V_4O_{10}$**	3M $Zn(CF_3SO_3)_2$	126 mAh g^{-1} at 0.2 A g^{-1}, 104 mAh g^{-1} at 0.6 A g^{-1}	70.3% after 5000 cycles at 2000 mA g^{-1}	[50]
**Zn/V_2O_5**	21M LiTFSI + 1M $Zn(CF_3SO_3)_2$	156 mAh g^{-1} at 1 A g^{-1}	80% after 2000 cycles at 2000 mA g^{-1}	[51]
**$Mg_xV_2O_5{\cdot}nH_2O$**	3M $Zn(CF_3SO_3)_2$	353 mAh g^{-1} at 0.05 A g^{-1}, 264 mAh g^{-1} at 1 A g^{-1}	97% after 2000 cycles at 5000 mA g^{-1}	[52]
**VO_2 (B)**	1 M $ZnSO_4$	110 mAh g^{-1} at 5C	40% after 200 cycles at 50 mA g^{-1}	[53]
